# stDyer-image improves clustering analysis of spatially resolved transcriptomics and proteomics with morphological images

**DOI:** 10.1093/bioinformatics/btag071

**Published:** 2026-02-15

**Authors:** Ke Xu, Xin Maizie Zhou, Lu Zhang

**Affiliations:** Department of Computer Science, Hong Kong Baptist University, Hong Kong, 999077, China; Department of Biomedical Engineering, Vanderbilt University, 2301 Vanderbilt Place, Nashville, Tennessee, 37235, United States; Department of Computer Science, Hong Kong Baptist University, Hong Kong, 999077, China

## Abstract

**Motivation:**

Spatially resolved transcriptomics (SRT) and spatially resolved proteomics (SRP) data enable the study of gene expression and protein abundances within their precise spatial and cellular contexts in tissues. Certain SRT and SRP technologies also capture corresponding morphology images, adding another layer of valuable information. However, few existing methods developed for SRT data effectively leverage these supplementary images to enhance clustering performance.

**Results:**

Here, we introduce stDyer-image, an end-to-end deep learning framework designed for clustering for SRT and SRP datasets with images. Unlike existing methods that utilize images to complement gene expression data, stDyer-image directly links image features to cluster labels. This approach draws inspiration from pathologists, who can visually identify specific cell types or tumor regions from morphological images without relying on gene expression or protein abundances. Benchmarks against state-of-the-art tools demonstrate that stDyer-image achieves superior performance in clustering. Moreover, it is capable of handling large-scale datasets across diverse technologies, making it a versatile and powerful tool for spatial omics analysis.

**Availability and implementation:**

The source code of stDyer-image and detailed tutorials are available at https://github.com/ericcombiolab/stDyer-image.

## 1 Introduction

Spatially resolved transcriptomic (SRT) and spatially resolved proteomics (SRP) measure spatial coordinates in addition to gene expression data or proteomics data, enabling the investigation of biological dynamics within their spatial contexts. Moreover, some SRT and SRP technologies (Chen *et al.* 2022, He *et al.* 2022, Hickey *et al.* 2023, Janesick *et al.* 2023) can simultaneously capture spatially annotated images, further enhancing the resolution and interpretability of the data. A critical task in SRT data analysis is identifying spatial domains where units exhibit similar gene expression patterns by considering the relationship between a target unit (either spots or cells, depending on the SRT technologies) and its spatial neighbors. Similarly, identifying cell types in SRT and SRP data is essential to facilitate downstream biological analysis. To address these tasks, various clustering methods (Hu *et al.* 2021, Zhao *et al.* 2021, Bao *et al.* 2022, Xu *et al.* 2022, Pham *et al.* 2023, Tang *et al.* 2023, Hu *et al.* 2024, 2025, Varrone *et al.* 2024) have been developed to incorporate spatial coordinates into the clustering process. However, only a few of them (Hu *et al.* 2021, Bao *et al.* 2022, Xu *et al.* 2022, Pham *et al.* 2023, Tang *et al.* 2023) utilize additional image information to further enhance clustering performance and improve biological insights.

SpaGCN (Hu *et al.* 2021) transforms morphological features of the units into artificial coordinates and integrates them with spatial coordinates to construct a graph that serves as input for its clustering algorithm. stLearn (Pham *et al.* 2023) uses matrix multiplication to combine a gene expression correlation matrix and a morphological similarity matrix for imputation. The imputed matrix is subsequently used to perform clustering analysis. SiGra (Tang *et al.* 2023) extracts image patches, flattens them into vectors, and reconstructs gene expression data from input to obtain augmented embeddings for clustering. DeepST (Xu *et al.* 2022) incorporates a morphological similarity matrix, a gene expression correlation matrix, and a spatial adjacency matrix to enhance gene expression data and generate embeddings for clustering. MUSE (Bao *et al.* 2022) uses a reconstruction loss to facilitate the mutual reconstruction of image and transcriptomic modalities, while a self-supervision loss ensures consistency of relative distance relationships between units across individual and joint modalities, optimizing embeddings for clustering analysis.

Although these methods have managed to combine transcriptome and image modalities for clustering analysis, a few limitations still remain. First, these methods often support only one or two specific technologies and applying them to custom datasets typically requires modifications or a thorough understanding of their source code due to insufficient documentation on input format specifications for the image modality. Second, existing tools that utilize the image modality are not scalable to large-scale datasets due to memory limitation. Last, gene expression level is not necessarily reflected by tissue color or shape, making it impossible to reliably “enhance” such genes based on images. For example, a gene may regulate the encoding of some transparent and tiny molecules that cannot be captured by images. Any attempt to modify or enhance the expression of such genes could therefore degrade the performance of downstream applications like clustering.

To address these limitations, we introduce stDyer-image, an end-to-end, scalable clustering approach for spatially resolved omics data. Unlike existing methods, stDyer-image directly associates the image modality with predicted labels to enhance clustering performance. This design draws inspiration from the practice of pathologists or trained clinicians, who can visually identify specific cell types or tumor regions by examining images, suggesting that images contain sufficient information to infer cluster labels. It also avoids introducing noise or irrelevant image features into the gene expression embeddings. Furthermore, stDyer-image uses a Gaussian Mixture Variational AutoEncoder (GMVAE), which directly optimizes predicted labels using objective functions that incorporate image similarities, further improving clustering accuracy and robustness. Additionally, stDyer-image is compatible with a wide range of spatially resolved omics technologies, including CosMx, Stereo-seq, 10x Xenium, and CODEX. Moreover, stDyer-image incorporates a mini-batch neighbor sampling strategy and supports multi-GPU training, enabling its application to large-scale datasets. We compared stDyer-image with eight state-of-the-art tools across five different technologies, demonstrating its superior performance and wide applicability.

## 2 Materials and methods

### 2.1 Data preprocessing

An SRT or SRP dataset with images should include three major components: (i) a gene expression matrix or protein abundance matrix with *m* genes or proteins for *n* units ($X\in R^{n\times m}$), (ii) spatial coordinates indicating the locations of units, and (iii) an image capturing the texture and density of units. Preprocessing of the gene expression matrix in SRT datasets was performed following standard conventions. Specifically, unless stated otherwise, we used Scanpy (Wolf *et al.* 2018) as follows: (i) removed feature names starting with “NegPrb” for the non-small-cell lung cancer dataset from CosMx, (ii) removed unlabeled units in the human breast cancer dataset from Xenium and units with spatial coordinates outside the image for all datasets, (iii) filtered units with zero RNA counts using [scanpy.pp.filter_cells(min_counts = 1)] and genes with zero RNA counts using [scanpy.pp.filter_genes(min_counts = 1)], (iv) selected the top 3000 highly variable genes using [scanpy.pp.highly_variable_genes], (v) performed library size normalization using [scanpy.pp.normalize_total], (vi) applied logarithmic transformation with [scanpy.pp.log1p], and (vii) standardized gene expression values to z-score using [scanpy.pp.scale]. All units after preprocessing are used for training.

**Figure 1:**
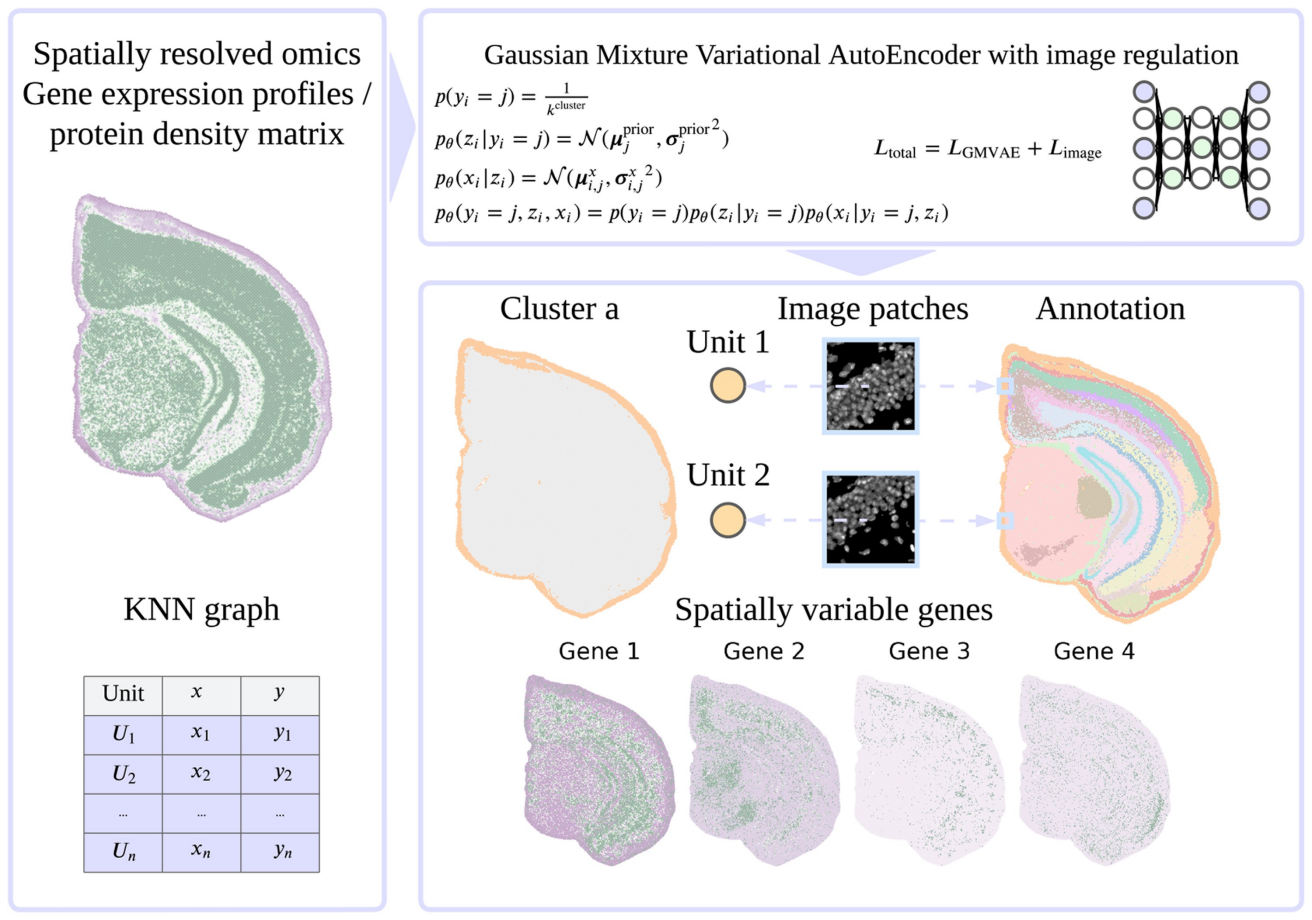
The workflow of stDyer-image. stDyer-image takes as input the gene expression profiles or protein abundance matrix along with a KNN graph. The GMVAE, incorporating a GAT, generates embeddings and cluster labels, which are optimized using loss functions that account for gene expression, protein abundance, and image data. The loss function involving gene expression profiles or protein abundance profiles maximizes the log-likelihood of the marginal distribution for modeling these profiles. This encourages neighboring units to have similar embeddings and cluster labels, relying solely on gene expression profiles or protein abundance profiles combined with spatial coordinates. The image-related loss function encourages units with similar image embeddings to share similar soft cluster labels. Additionally, stDyer-image can identify spatially variable genes using integrated gradient analysis.

For images, we selected the highest-resolution image (typically with the .tif or .tiff suffix) for each dataset and cropped them into 250 × 250-pixel patches. We determined that a patch size of 250 × 250 pixels adequately represent the local environment of a cell and its neighboring cells (Fig. 1). For single-channel images, we converted them into three channels using [np.broadcast_to] to facilitate feature extraction. Another preprocessing step is the alignment of the coordinates of SRT to the morphological image. This is necessary when the two data sources are generated in separate coordinate systems. Alignment involves applying a provided affine transformation matrix to the spatial coordinates, performing operations such as translation, rotation, and scaling to map them onto the image space. This ensures that each unit from SRT is accurately associated with its visual context in the tissue image. The result is validated by visually overlaying the transformed coordinates onto the image (Fig. S3, available as supplementary data at *Bioinformatics* online). Specifically, for the mouse brain dataset from Stereo-seq, we replaced the function “np.round” with “np.floor” function inside the “read_bgi_agg” function from the Spateo package (Qiu *et al.* 2024) and set the parameter “prealigned” to “False” as well as the parameter “binsize” to “1” to retrieve information necessary for alignment. Subsequently, we applied the “spateo.segmentation.refine_alignment” function, specifying “mode” = “rigid,” to align the image accurately. For the human breast cancer dataset from Xenium, we used the provided affine matrix for alignment using the “skimage.transform.AffineTransform” and “skimage.transform.warp” functions. For the NSCLC dataset (CosMx), spatial coordinates provided in the “spatial_global” attribute aligned with the image without additional processing. Images from the human intestine (CODEX) did not require additional processing as well.

### 2.2 Image feature processing

Image patches were fed into the ResNet18 model pre-trained on morphological image datasets (Ciga *et al.* 2022), and we removed the last fully connected layer to obtain image feature embeddings. Since stDyer-image was trained using a mini-batch strategy, K-nearest image neighbors were identified for each unit within the same batch based on the cosine distance metric. These image neighbors were then incorporated into the computation of image-related loss during training (see Section 4.4).

### 2.3 The network structure of stDyer-image and associated probabilistic models

The network architecture of stDyer-image and its associated probabilistic models mirror those of stDyer and are briefly described here. The GMVAE network (Jiang *et al.* 2017) consists of two main components: an encoder, which models the inference process, and a decoder, which models the generative process. The encoder is a GAT (Petar Veličković *et al.* 2018) that dynamically aggregates gene expression profiles or protein abundance profiles from the neighbors of a given unit in a graph. The decoder reconstructs the gene expression profiles or protein abundance profiles of the target unit. The generative process, parameterized by θ, in stDyer-image is represented as follows:


(1) $$\begin{array}{c} p(y_{i}=j)=\frac{1}{k^{\text{cluster}}},j=1,2,\ldots ,k^{\text{cluster}} \end{array}$$

(2) $$\begin{array}{c} p_{\theta }(z_{i}|y_{i}=j)=N(\mu_{j}^{\text{prior}},\sigma_{j}^{\;\text{prior}}{\;}^{2}),j=1,2,\ldots ,k^{\text{cluster}} \end{array}$$

(3) $$\begin{array}{c} p_{\theta }(x_{i}|z_{i})=N(\mu_{i,j}^{x},\sigma_{i,j}^{x}{\;}^{2}),j=1,2,\ldots ,k^{\text{cluster}} \end{array}$$

(4) $$\begin{array}{c} p_{\theta }(y_{i}=j,z_{i},x_{i})=p(y_{i}=j)p_{\theta }(z_{i}|y_{i}=j)p_{\theta }(x_{i}|y_{i}=j,z_{i}),\\ \;j=1,2,\ldots ,k^{\text{cluster}} \end{array}$$

Let $x_{i}$, $y_{i}$ and $z_{i}$ denote the gene expression profile or protein abundance profile, cluster label, and latent embedding from the *j*th mode of GMM for the target unit *i*, respectively. The parameters $\mu_{j}^{\text{prior}}$ and $\sigma_{j}^{\text{prior}}$ represent the mean and standard deviation of the prior distribution for the *j*th Gaussian mixture. The prior distribution is set to be uniform, following a common practice in GMVAE-based clustering method (Jiang *et al.* 2017). A uniform prior reflects the absence of any initial bias toward specific clusters, allowing cluster assignment to be learned purely from the data during training. This choice encourages balanced exploration of all *k* clusters and prevents the model from being influenced by preconceived assumptions about cluster proportions. Similarly, $\mu_{i,j}^{x}$ and $\sigma_{i,j}^{x}$ denote the mean and standard deviation of the output distribution (i.e. multivariate Gaussian distribution). Using a multivariate Gaussian distribution as the output distribution stabilizes the training process by avoiding direct reconstruction of raw gene expression counts or protein abundance profiles. Furthermore, using continuous distribution enables stDyer-image to adapt to integer inputs that require normalization to floating-point numbers. Notably, the output distribution is unit-wise, meaning that each unit has its own unique multivariate Gaussian distribution. This approach is different from other methods that utilize a single negative binomial distribution to represent all gene expression values of each unit. Consequently, the output distribution does not influence the sparsity of the reconstructed values but instead defines the output values as continuous. The inference process, parameterized by ϕ, is formulated as follows:


(5) $$\begin{array}{c} q_{\phi }(y_{i}=j|x_{i},{\left\{x_{i}\right\}}^{\text{neigh}})={\hat{Y}}_{i}(j),j=1,2,\ldots ,k^{\;\text{cluster}} \end{array}$$

(6) $$\begin{array}{c} q_{\phi }(z_{i}|x_{i},{\left\{x_{i}\right\}}^{\text{neigh}},y_{i}=j)=N(\mu_{i,j}^{\text{post}},\sigma_{i,j}^{\text{post}}{\;}^{2}),\\ \;j=1,2,\ldots ,k^{\text{cluster}} \end{array}$$

(7) $$\begin{array}{c} q_{\phi }(y_{i}=j,z_{i}|x_{i},{\left\{x_{i}\right\}}^{\text{neigh}})=q_{\phi }(y_{i}=j|x_{i},{\left\{x_{i}\right\}}^{\text{neigh}})\\ \cdot q_{\phi }(z_{i}|x_{i},{\left\{x_{i}\right\}}^{\text{neigh}\;},y_{i}=j),\\ \;j=1,2,\ldots ,k^{\text{cluster}} \end{array}$$

where ${\hat{Y}}_{i}(j)$ denotes the predicted probability that the target unit *i* belongs to the *j*th mode of GMM, and ${\left\{x_{i}\right\}}^{\text{neigh}}$ represents gene expression profiles or protein abundance profiles of the neighbors of unit *i*. The cluster assignment vector ${\hat{Y}}_{i}(j)$ is inferred from the input $x_{i}$ and ${\left\{x_{i}\right\}}^{\text{neigh}}$ using GAT and is gradually refined during training. $\mu_{j}^{\text{post}}$ and $\sigma_{j}^{\text{post}}$ denote the mean and standard deviation of the posterior distribution for the *j*th Gaussian mixture.

### 2.4 The objective function of stDyer-image

The objective function of stDyer-image consists of an image-related objective function and the original objective function of stDyer. The image-related objective function encourages units with similar image embeddings to share similar soft cluster labels. On the other hand, the objective function of stDyer encourages neighboring units to have similar embeddings and cluster labels based solely on gene expression or protein abundance data combined with spatial coordinates. This is achieved by maximizing the log-likelihood of the marginal distribution used to model the gene expression or protein abundance data.

The objective function of stDyer-image can be written as:


(8) $$\underset{\theta ,\phi }{\max}\sum_{i=1}^{n}\left(-L_{\text{image}}^{i}+\text{log}\;p_{\theta }(x_{i})-{\text{JS}}^{i}\right)$$

where $L_{\text{image}}$ is the image-related loss function of unit *i*, $x_{i}$ refers to the gene expression profiles or protein abundance profile of unit *i* and ${\text{JS}}^{i}$ denotes the Jensen-Shannon divergence of unit *i*.

The image-related loss function is computed for each unit *i* with its *k* image neighbors (default *k *= 8) within the mini-batch. These image neighbors are the units whose image embeddings, derived from their associated image patches, are most similar to that of unit *i*. Unit *i* and its image neighbors are encouraged to have higher probabilities for their corresponding soft labels.


(9) $$L_{\text{image}}^{i}=\sum_{j=1}^{k}-\text{log}\;\left({\hat{Y}}_{i}\cdot {\hat{Y}}_{j}\right)$$

### 2.5 Evaluation metrics

We used the Adjusted Rand Index to evaluate the performance of clustering results (Hubert and Arabie 1985). The ARI score measures the agreement between the ground truth cluster labels and the predicted cluster labels, with scores ranging from −1 to 1. For evaluation, all methods are required to predict the same number of clusters as the ground truth. A higher ARI score indicates better clustering performance. Given the ground truth cluster $A=(a_{1},a_{2},\ldots ,a_{t})$ and the predicted cluster $B=(b_{1},b_{2},\ldots ,b_{p})$, the ARI is defined as follows:


(10) $$\mathrm{ARI}=\frac{\sum_{i,j}\left(\begin{array}{c} c_{i,j}\\ 2 \end{array}\right)-\left[\sum_{i}\left(\begin{array}{c} a_{i}\\ 2 \end{array}\right)\sum_{j}\left(\begin{array}{c} b_{j}\\ 2 \end{array}\right)\right]/\left(\begin{array}{c} n\\ 2 \end{array}\right)}{\frac{1}{2}\left[\sum_{i}\left(\begin{array}{c} a_{i}\\ 2 \end{array}\right)+\sum_{j}\left(\begin{array}{c} b_{j}\\ 2 \end{array}\right)\right]-\left[\sum_{i}\left(\begin{array}{c} a_{i}\\ 2 \end{array}\right)\sum_{j}\left(\begin{array}{c} b_{j}\\ 2 \end{array}\right)\right]/\left(\begin{array}{c} n\\ 2 \end{array}\right)}$$

where $c_{i,j}=|a_{i}\cap b_{j}|$, and *n* is the number of units.

## 3 Results

### 3.1 Workflow of stDyer-image

stDyer-image builds on the GMVAE (Jiang *et al.* 2017), integrating it with a Graph ATtention network (GAT) (Petar Veličković *et al.* 2018) to jointly generate embeddings and cluster labels, similar to stDyer (Xu *et al.* 2025). The GMVAE processes inputs such as gene expression profiles or protein abundance profiles, along with a K-nearest neighbor (KNN) graph (Fig. 1). The KNN graph can be constructed based on spatial coordinates, gene expression profiles, or protein abundance matrix, depending on whether the task involves delineating spatial domains or identifying cell types. The major difference between stDyer-image and stDyer is that stDyer-image incorporates image patches into an image-related loss function to improve clustering performance.

The loss functions of stDyer-image comprise the original function from stDyer and an additional image-related loss function. The image-related loss function involves the soft probabilities of predicted cluster labels and the cosine similarity between the soft cluster labels of a unit and its image neighbors. Image neighbors are defined as units with similar image patches to the target unit. This image-related loss establishes a connection between the soft cluster label of a unit and its image neighbors, encouraging units with similar image patches to share similar soft cluster labels. The loss function from stDyer includes the log-likelihood of the marginal distribution for modeling gene expression data or protein abundance data, as well as the reconstruction loss of these data. This component promotes similar embeddings and soft cluster labels among neighboring units, relying solely on gene expression or protein abundance data. By integrating the image-related loss, stDyer-image further enhances clustering performance by incorporating image-derived information.

### 3.2 stDyer-image identifies tumor on the NSCLC dataset from CosMx technology

**Figure 2:**
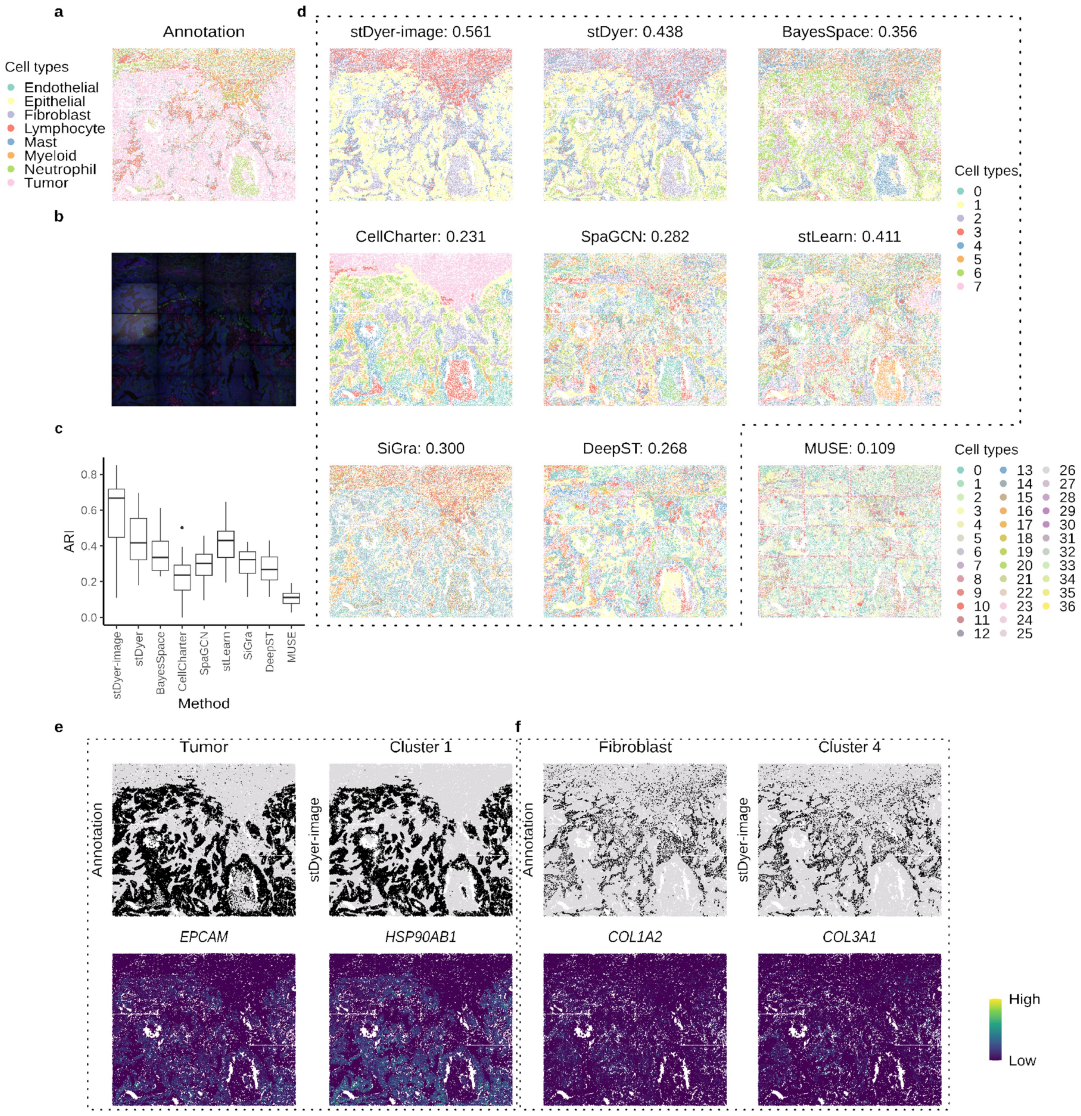
Performance of stDyer-image on a NSCLC dataset from CosMx technology. (a) Visualization of the annotation for the NSCLC dataset. (b) Morphology image of the NSCLC dataset. (c) Boxplot of ARI scores for nine methods evaluated across 20 slices of the NSCLC dataset. (d) Visualization and the average ARI scores across 20 slices for cell type clustering using different methods on the NSCLC dataset. (e) Visualization of the annotation for tumor regions, the prediction of cluster 1 by stDyer-image, and two selected SVGs for cluster 1. (f) Visualization of the annotation for fibroblast regions, the prediction of cluster 4 by stDyer-image, and two selected SVGs for cluster 4.

We evaluated the performance of stDyer-image on a human non-small cell lung cancer (NSCLC) dataset (He *et al.* 2022) generated using CosMx technology. This dataset consists of 20 slices (Fig. 2a) and their associated images (Fig. 2b), resulting in a total of 87 606 units. These slices are annotated with eight cell types (Fig. 2a) by referring to He *et al.* (2022). These slices are arranged in a 5×4 grid to accommodate a large area of tissue. We benchmarked stDyer-image against eight state-of-the-art methods for clustering analysis on SRT data: BayesSpace, CellCharter, stDyer, SpaGCN, stLearn, SiGra, DeepST, and MUSE, using adjusted rand index (ARI; Section 2), Silhouette score (SI; Section 2), and Fowlkes-Mallows index (FMI) as the evaluation metrics. Among these, BayesSpace, CellCharter, and stDyer are scalable to large datasets but are incapable of using images, whereas the remaining methods are capable of utilizing images for clustering analysis. Importantly, stDyer-image, stDyer, BayesSpace, CellCharter, and SiGra can analyze all 20 slices simultaneously and provide consistent predictions across slices, while the other methods are limited to processing one slice at a time. It is worth noting that 20 slices have a unified spatial coordinate system. However, stDyer-image can still obtain unified cluster labels across multiple slices even if their coordinates are not unified (Supplementary Methods, available as supplementary data at *Bioinformatics* online). We reported the single-slice ARI scores of each method in the box plot (Fig. 2c) and observed that stDyer-image performed the best on the NSCLC dataset with an average ARI of 0.561 across all 20 slices (Fig. 2d). stDyer-image also obtained the highest SI on gene expression of −0.019, the highest SI on embedding of 0.323, and the highest FMI of 0.717 compared to other methods (Table S1, available as supplementary data at *Bioinformatics* online). stDyer-image demonstrated superior performance in predicting tumor regions while stDyer, BayesSpace, and CellCharter tended to split them into multiple parts. While SiGra identified the entire tumor regions, it failed to accurately identify neutrophil cells located in proximity. SpaGCN, stLearn, and DeepST struggled to produce consistent cluster labels across slices. MUSE did not accept a fixed cluster number as input and produced an excessive number of clusters. Besides, MUSE performed poorly on slice boundaries.

We further evaluated the scalability of image-utilizing methods by sampling 100 000, 200 000, 500 000, and 1 000 000 units from the NSCLC dataset (Fig. S7, available as supplementary data at *Bioinformatics* online). Each sampled dataset was treated as a single slice rather than 20 slices in the original dataset. Methods with GPU support were run on an Nvidia A100 GPU card with 80G graphics memory, whereas methods without GPU support were run on a server with two sets of Intel Xeon Gold 6330 CPU (@ 2.0 GHz, 56 cores/112 threads), 2 TB host memory, and 900 GB of virtual memory. We observed that stDyer-image was the only method that could process 1 000 000 units. In contrast, all other methods failed to process such a large dataset due to insufficient graphics memory or host memory. In addition, stDyer-image consistently outperformed other methods in terms of running time across all sampled dataset sizes.

To explore whether the domains identified by stDyer-image facilitate the detection of biologically relevant biomarkers, we ranked the top 50 spatially variable genes (SVGs) with the highest integrated gradient (IG) values for each cluster (Section 2). For visualization purposes, we selected two genes that strongly aligned with cluster 1 and 4, respectively (Fig. 2e and f, Fig. S4, available as supplementary data at *Bioinformatics* online). For instance, cluster 1, which corresponded to the tumor region in the annotation (Fig. 2e), included *EPCAM* as one of its SVGs. The protein EPCAM is known to be widely present in NSCLC tumor regions but not in normal Lung tissue (Pak *et al.* 2012). Additionally, another SVG for cluster1, *HSP90AB1*, was highly expressed in NSCLC tumor tissue and is associated with poor prognosis in lung adenocarcinoma patients (Minghui *et al.* 2016). Cluster 4, corresponding to fibroblasts in the annotation (Fig. 2f), included *COL1A2* and *COL3A1* as highly expressed SVGs. The cluster associated with these genes was classified as matrix cancer-associated fibroblast by Cords *et al.* (2023).

### 3.3 stDyer-image recognizes smooth laminar layers on the mouse brain dataset from stereo-seq technology

**Figure 3:**
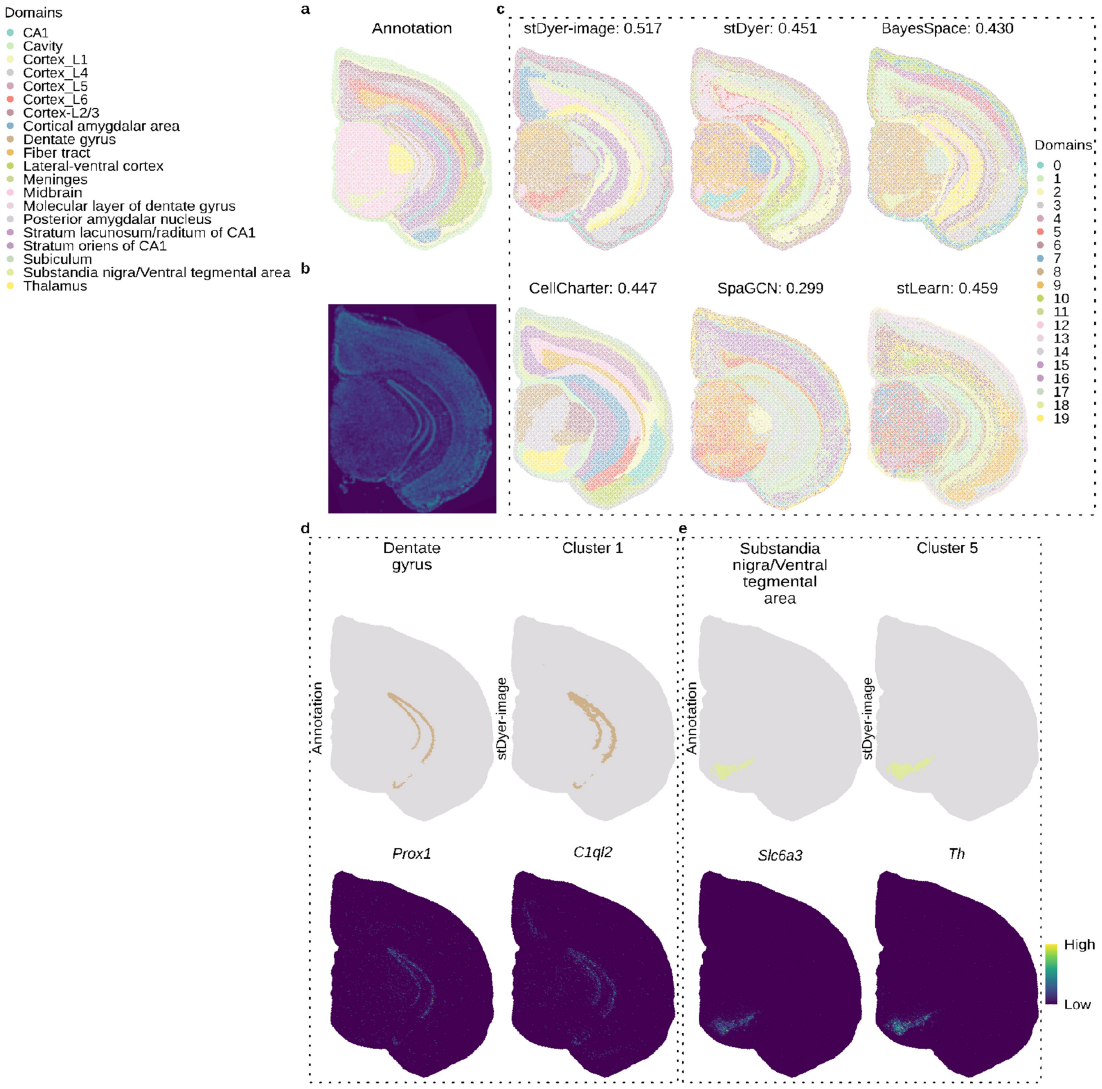
Performance of stDyer-image on a mouse brain dataset from Stereo-seq technology. (a) Visualization of the annotation for mouse brain tissue. (b) Morphology image of mouse brain tissue. (c) Visualization and ARI scores of various methods for spatial domain clustering on mouse brain tissue. (d) Visualization of the annotation for Dentate gyrus, the prediction of cluster 1 by stDyer-image, and the two selected SVGs for cluster 1. (e) Visualization of the annotation for Substandia nigra/Ventral tegmental area, the prediction of cluster 5 by stDyer-image, and the two selected SVGs for cluster 5.

The mouse brain dataset (Chen *et al.* 2022), generating using Stereo-seq technology, comprises 38 746 units and 23 905 genes, with 20 annotated spatial domains (Fig. 3a and b) based on markers and anatomic annotation (Chen *et al.* 2022). We benchmarked stDyer-image against stDyer, BayesSpace, CellCharter, SpaGCN and stLearn. Among these methods, stDyer-image achieved the highest ARI score of 0.517 (Fig. 3c). Other methods (SiGra, DeepST, and MUSE) failed to run on this large dataset due to out of memory (OOM) errors. stDyer-image produced more spatially coherent layer predictions compared to stDyer, BayesSpace, SpaGCN, and stLearn. While CellCharter achieved even smoother predictions, it failed to identify thin layers such as Dentate gyrus. stDyer-image obtained the highest SI score on gene expression of −0.019, the highest SI score on embeddings of 0.146 (Table S2, available as supplementary data at *Bioinformatics* online), and the highest score of 0.557 on FMI. We performed a similar SVG analysis, identifying SVGs associated with specific clusters based on their IG values (Fig. 3d and e, Fig. S5, available as supplementary data at *Bioinformatics* online). For example, Dentate gyrus was identified as cluster 1 (Fig. 3d), with the SVG *Prox1* (Fig. 3d) playing a crucial role in the maintenance and maturation of Dentate gyrus granule cells (Lavado *et al.* 2010). Additionally, the SVG *C1ql2* was found to be highly expressed in Dentate gyrus (Iijima *et al.* 2010). In the Ventral tegmental area, the SVG *Slc6a3* (Fig. 3e), encoding the dopamine transporter, was associated with the rewarding function of this region (Fitzgerald and Day 2025). Another SVG, *Th* (Fig. 3e), a known marker of dopaminergic neurons (Fitzgerald and Day 2025), was also identified to be highly expressed in the Ventral tegmental area.

### 3.4 stDyer-image deciphers clear laminar structures on the human intestine dataset from CODEX technology

**Figure 4:**
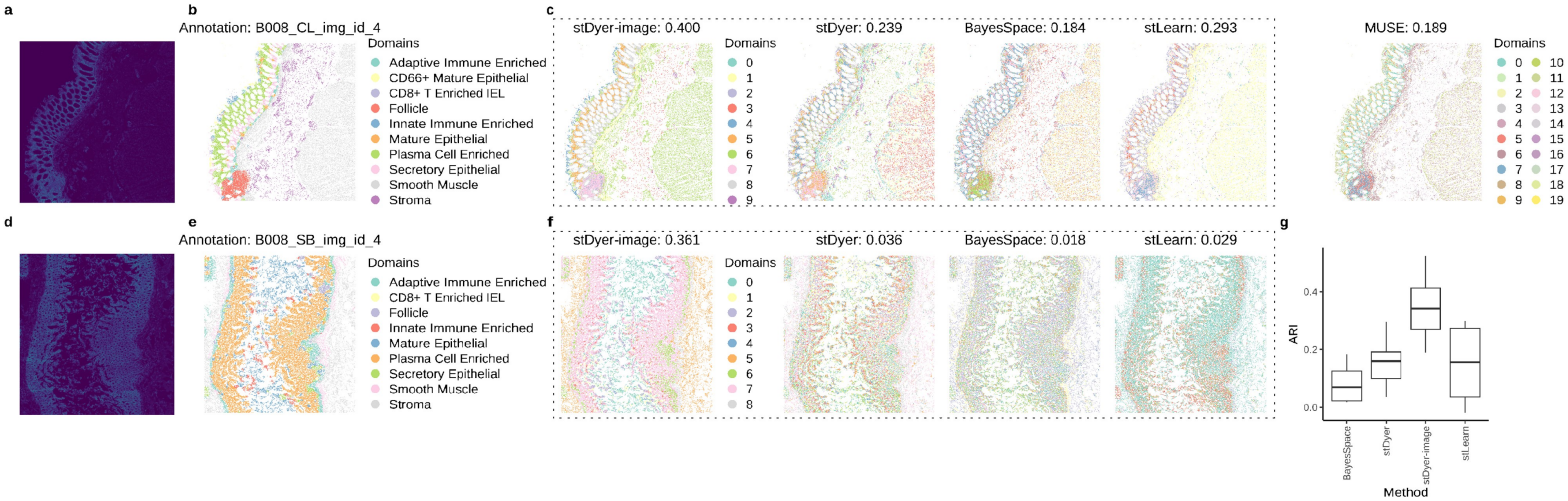
Performance of stDyer-image on a human intestine dataset from CODEX technology. (a) Morphology image of the fourth slice from colon tissue of the B008 donor. (b) Visualization of the annotation for the fourth slice from colon tissue of the B008 donor. (c) Visualization and ARI scores of different methods for spatial domain clustering on the 4th slice from colon tissue of B008 donor. (d) Morphology image of the fourth slice from small bowel tissue of the B008 donor. (e) Visualization of the annotation for the fourth slice from small bowel tissue of the B008 donor. (f) Visualization and ARI scores of different methods for spatial domain clustering on the fourth slice from small bowel tissue of the B008 donor. (g) Boxplot of ARI scores for four methods evaluated across all eight slices from the B008 donor.

Given the similarity in format between SRP data and SRT data, we applied stDyer-image to a human intestine dataset (Hickey *et al.* 2023) from CODEX technology. This dataset contained 64 slices from 8 donors, with each slice characterized by measurements of 47 proteins. For each donor, 8 slices were included, with four from the colon and four from the small intestine. These slices are non-adjacent and spatially separated. We first benchmarked stDyer-image on two slices, one from the colon (Fig. 4a and b) and one from the small intestine (Fig. 4d and e). For the colon tissue, stDyer-image achieved an ARI score of 0.400 (Fig. 4c), outperforming the second-best method, stLearn (ARI = 0.293). Compared to stLearn, stDyer-image identified more heterogeneity in the leftmost layers. For example, cluster 1 predicted by stDyer-image corresponded to a region that aligned with Adaptive Immune Enriched domain. Additionally, stDyer-image uniquely identified the distinct left and right regions within the Smooth Muscle domain, whereas other methods erroneously mixed the left region with the Stroma domain (Fig. 4f). For the small intestine tissue, stDyer-image achieved an ARI score of 0.361, outperforming the second-best method stDyer (ARI = 0.036). All other methods failed to delineate continuous domains and instead mixed different domains together. DeepST and MUSE failed to process this slice (unit number: 78 213) due to OOM errors. We further benchmarked stDyer-image on all 8 slices (with the unit number ranging from 5829 to 78 213) from donor B008. SpaGCN was incompatible with datasets because it requires at least 50 features to perform PCA internally, while CellCharter could not be applied because the dataset provided normalized values rather than raw counts. SiGra, DeepST, and MUSE failed due to OOM errors. stDyer-image achieved the highest average ARI score of 0.346, outperforming stDyer (ARI = 0.157), stLearn (ARI = 0.152), and BayesSpace (ARI = 0.082) (Fig. 4g). stDyer-image achieved a low average SI score on gene expression of 0.006, while stLearn achieved the highest average SI score on gene expression of 0.315 (Table S3, available as supplementary data at *Bioinformatics* online). We also evaluated the SI score on gene expression with annotation labels and obtained its SI score as −0.003, suggesting that it may be impossible to obtain high SI scores on gene expression and ARI scores simultaneously on this dataset. We also checked the average SI score on embeddings of stDyer-image and stDyer, respectively. stDyer-image had the highest average SI score on embeddings of 0.010 (Table S3, available as supplementary data at *Bioinformatics* online) compared to stDyer with a score of −0.044, indicating its embeddings have better separateness. stDyer-image also obtained the highest FMI of 0.582 compared to other methods.

## 4 Discussion

During benchmarking of existing clustering methods designed for spatially resolved omics data, we observed that the scalability of most methods is limited. Some are constrained by the available GPU memory since they lack a mini-batch strategy to handle large-scale datasets. Others suffer from inefficient optimization, with excessive IO operations related to image processing that significantly slow down clustering analysis. In contrast, stDyer-image is designed to be more memory-efficient, using a mini-batch strategy to process large-scale datasets. Additionally, IO operations are minimized in stDyer-image to speed up clustering analysis when images are used as input, especially when temporary file storage relies on slow-speed devices such as hard disk drives.

stDyer-image is compatible with data from various technologies, unlike other methods that utilize images but are restricted to one or two technologies. Running those methods on unsupported datasets often requires modification to their code or the dataset structure. This process can be challenging due to a lack of detailed documentation on the required input format. In contrast, we have provided comprehensive descriptions of the input format, facilitating the use of stDyer-image on customized datasets. Additionally, a user-friendly interface allows users to run stDyer-image on their datasets with just a few lines of code. Unlike many other methods that require raw counts as input, stDyer-image can also process normalized data, offering greater flexibility and ease of use. Furthermore, stDyer-image supports multi-GPU acceleration, significantly enhancing its capability to analyze large-scale datasets efficiently. These features make stDyer-image a robust and accessible tool for spatial omics data analysis.

## Supplementary Material

btag071_Supplementary_Data

## Data Availability

The NSCLC dataset (He *et al.* 2022) from CosMx technology is available in https://staging.nanostring.com/products/cosmx-spatial-molecular-imager/ffpe-dataset/nsclc-ffpe-dataset/. The expression profiles and image of mouse brain dataset (Chen *et al.* 2022) from Stereo-seq technology can be directly downloaded from https://ftp.cngb.org/pub/SciRAID/stomics/STDS0000058/stomics/Mouse_brain.h5ad and https://ftp.cngb.org/pub/SciRAID/stomics/STDS0000058/Image/Mouse_brain_Adult.tif, respectively. The human breast cancer dataset (Janesick *et al.* 2023) from 10x Xenium technology was downloaded from https://www.10xgenomics.com/products/xenium-in-situ/preview-dataset-human-breast. The human intestine dataset (Hickey *et al.* 2023) from CODEX technology are accessible at https://datadryad.org/stash/dataset/doi:10.5061/dryad.76hdr7t1p. The corresponding annotation can be accessed at https://datadryad.org/stash/dataset/doi:10.5061/dryad.pk0p2ngrf. The reproducible materials are available on Zenodo: https://doi.org/10.5281/zenodo.15243986. The source code of stDyer-image is publicly available at GitHub: https://github.com/ericcombiolab/stDyer-image.
