# Supplementary material for: stDyer-image improves clustering analysis of spatially resolved transcriptomics and proteomics with morphological images
Source: Bioinformatics. 2026 Feb 15;42(3):btag071. doi: 10.1093/bioinformatics/btag071 (PMC12960910; doi:10.1093/bioinformatics/btag071)
Supplement: btag071_Supplementary_Data [file btag071_supplementary_data.pdf]

Supplementary Results

stDyer-image detects proliferative invasive tumor on a human breast cancer dataset from 10x Xenium technology

We also analyzed a human breast cancer dataset (Janesick et al., 2023) from 10x Xenium technology, consisting of 159,226 units and 313 genes, annotated with 19 cell types (Figure S1a and b) that are transferred from scFFPE-seq annotations (Janesick et al., 2023). We benchmarked stDyer-image against stDyer, BayesSpace, CellCharter, SpaGCN and stLearn. stDyer-image achieved the highest ARI score of 0.516 (Figure S1c) and the second-highest SI score on gene expression of -0.002 compared to stLearn with the highest SI score on gene expression of 0.005 (Table S4). For the SI score on embeddings, stDyer-image had the highest score of 0.166 compared to stDyer (0.131) and CellCharter (0.016) (Table S4). stDyer-image achieved the highest FMI of 0.582 as well. SiGra, DeepST, and MUSE still failed to process this large dataset due to OOM errors. Further comparisons were made across Stroma and Invasive Tumor clusters, which represent the two largest annotated clusters in the dataset (41,422 units and 34,374 units, respectively, out of 159,226 units). stDyer-image, stDyer, BayesSpace, and SpaGCN identified the most extensive region for Stroma. While stLearn performed well overall, it introduced segmentation artifacts at the right center (Figure S2). CellCharter, on the other hand, identified more units belonging to Stroma in the central region but divided the cluster into smaller clusters at the top and bottom. For Invasive Tumor, only stDyer-image and stLearn predicted 29,347 and 26,304 units in the cluster 2 and 13, respectively. Other methods predicted units fewer than 15,000 units in their largest cluster matched with Invasive Tumor.

We performed IG analysis (Figure S6) and found that Invasive Tumor (Figure S1d) corresponded to cluster 2 (Figure S1e), which was associated with the SVG *FASN* (Figure S1f). *FASN* is known to overexpress in human breast cancer, serving as a marker of poor prognosis (Vanauberg et al., 2023). It mediates changes in certain fatty acids that promote tumor migration (Xu et al., 2021). Similarly, *CENPF*, the SVG for cluster 12, was found to be associated with Proliferative Invasive Tumor. Overexpression of this gene has been linked to tumor bone metastasis in breast cancer (Sun et al., 2019) and can result in chromosomal instability (O'Brien et al., 2007), and it is also indicative of poor prognosis (Sun et al., 2019; O'Brien et al., 2007). Additionally, the SVGs *KRT15* and *ACTA2* were identified as being associated with clusters 14 and 18, corresponding to Myoepi KRT15+ and Myoepi ACTA2+ regions, respectively, as indicated in the annotations.

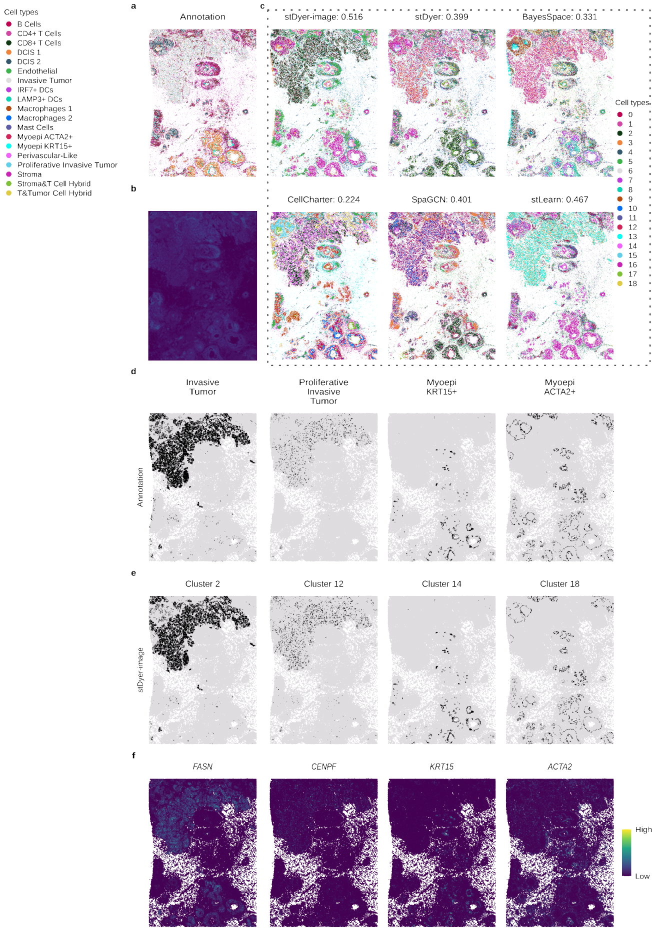

Fig. S1. Performance of stDyer-image on a human breast cancer dataset from 10x Xenium technology. (**a**) Visualization of the annotation for the human breast cancer dataset. (**b**) Morphology image of the human breast cancer dataset. (**c**) Visualization and ARI scores of different methods for cell type clustering on the human breast cancer dataset. (**d**) Visualization of the annotation for Invasive Tumor, Proliferative Invasive Tumor, Myoepi KRT15+, and Myoepi ACTA2+. (**e**) Visualization of predictions for clusters 2, 12, 14, and 18 from stDyer-image. (**f**) Visualization of SVG associated with clusters 2, 12, 14, and 18 from stDyer-image.

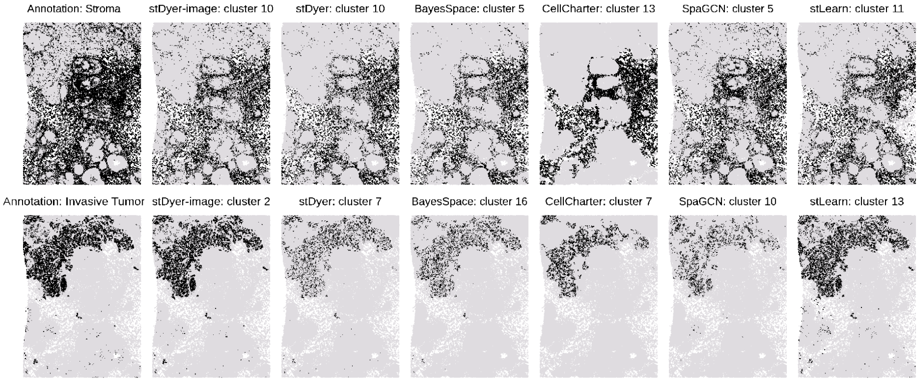

Fig. S2: The predictions of Stroma and Invasive Tumor of each method.

Metrics comparison

Method	Silhouette score (gene expression)	Silhouette score (embeddings)	Fowlkes-Mallows index
Annotation	0.020	N/A	1
BayesSpace	-0.075	N/A	0.515
CellCharter	-0.087	0.073	0.367
DeepST	-0.039	-0.014	0.213
MUSE	-0.045	-0.117	0.181
SiGra	-0.079	0.017	0.483
SpaGCN	-0.039	N/A	0.202
stDyer	-0.066	0.276	0.581
stDyer-image	-0.019	0.323	0.717
stLearn	-0.031	N/A	0.219

Table S1. Metrics results for NSCLC dataset from CosMx technology.

Method	Silhouette score (gene expression)	Silhouette score (embeddings)	Fowlkes-Mallows index
Annotation	-0.027	N/A	1
BayesSpace	-0.088	N/A	0.478
CellCharter	-0.048	0.139	0.496
SpaGCN	-0.238	N/A	0.364
stDyer	-0.046	0.075	0.497
stDyer-image	-0.039	0.146	0.557
stLearn	-0.122	N/A	0.503

Table S2. Metrics results for mouse brain dataset from Stereo-seq technology.

Method	Silhouette score (gene expression)	Silhouette score (embeddings)	Fowlkes-Mallows index
Annotation	-0.003	N/A	1
BayesSpace	0.009	N/A	0.289
stDyer	-0.068	-0.044	0.331
stDyer-image	0.006	0.010	0.534
stLearn	0.315	N/A	0.464

Table S3. Metrics results for human intestine dataset from CODEX technology.

Method	Silhouette score (gene expression)	Silhouette score (embeddings)	Fowlkes-Mallows index
Annotation	-0.014	N/A	1
BayesSpace	-0.032	N/A	0.425
CellCharter	-0.053	0.016	0.319
SpaGCN	-0.029	N/A	0.480
stDyer	-0.011	0.131	0.488
stDyer-image	-0.002	0.166	0.582
stLearn	0.005	N/A	0.539

Table S4. Metrics results for human breast cancer dataset from 10x Xenium technology.

Verification demonstration of image alignment

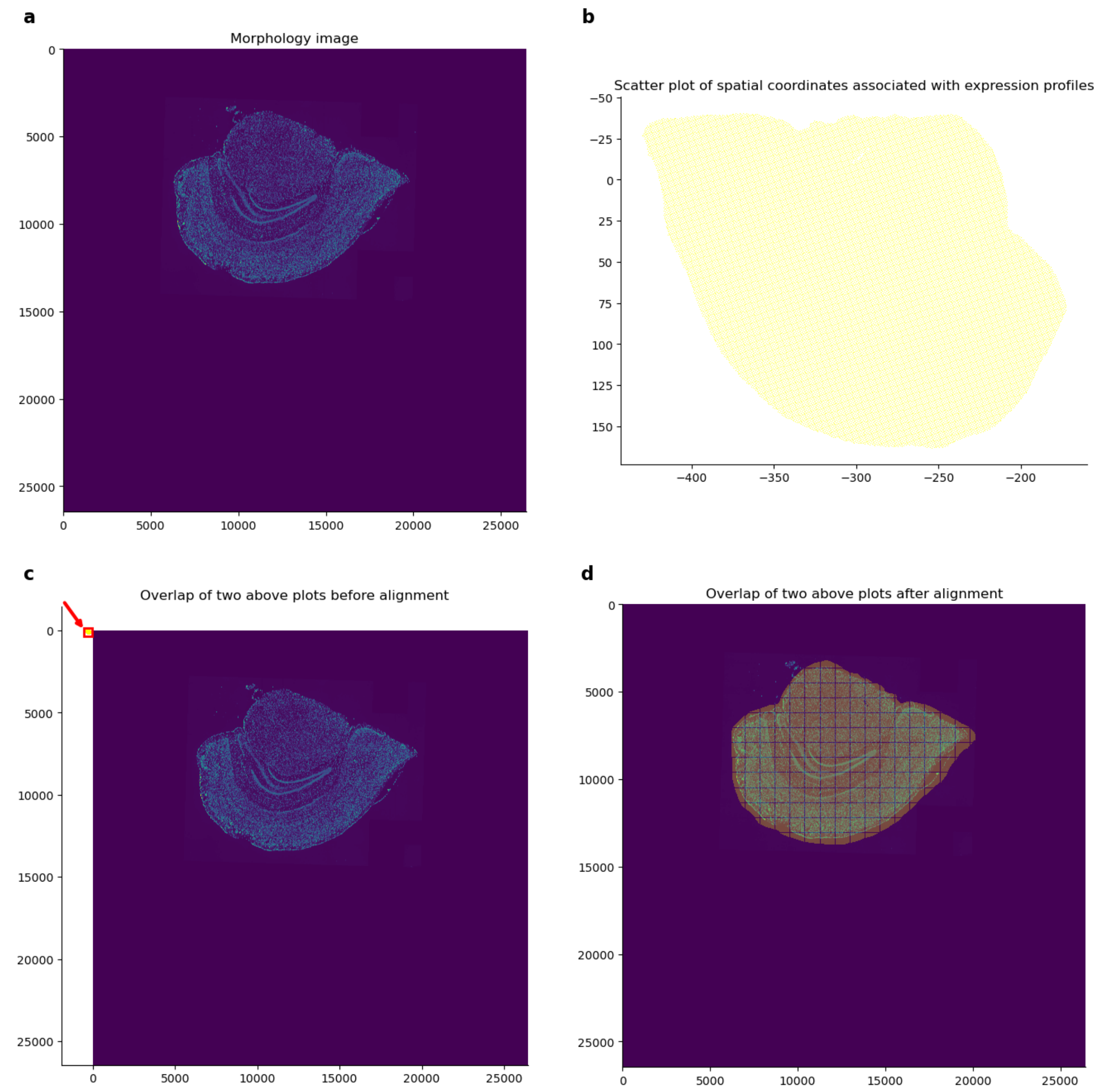

Fig. S3. The morphology image (**a**) and coordinates scatter plot of spatial transcriptomics (**b**) of mouse brain from Stereo-seq. The overlay of **a** and **b** before alignment (**c**), and the overlay of **a** and **b** after alignment (**d**).

Top spatially variable genes on each dataset

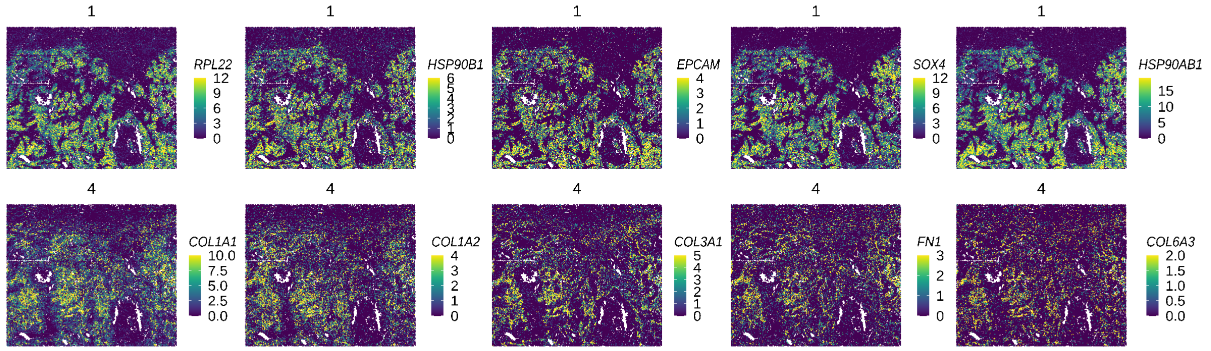

Fig. S4. Top 5 SVGs of cluster 1 and 4 for NSCLC dataset from CosMx technology.

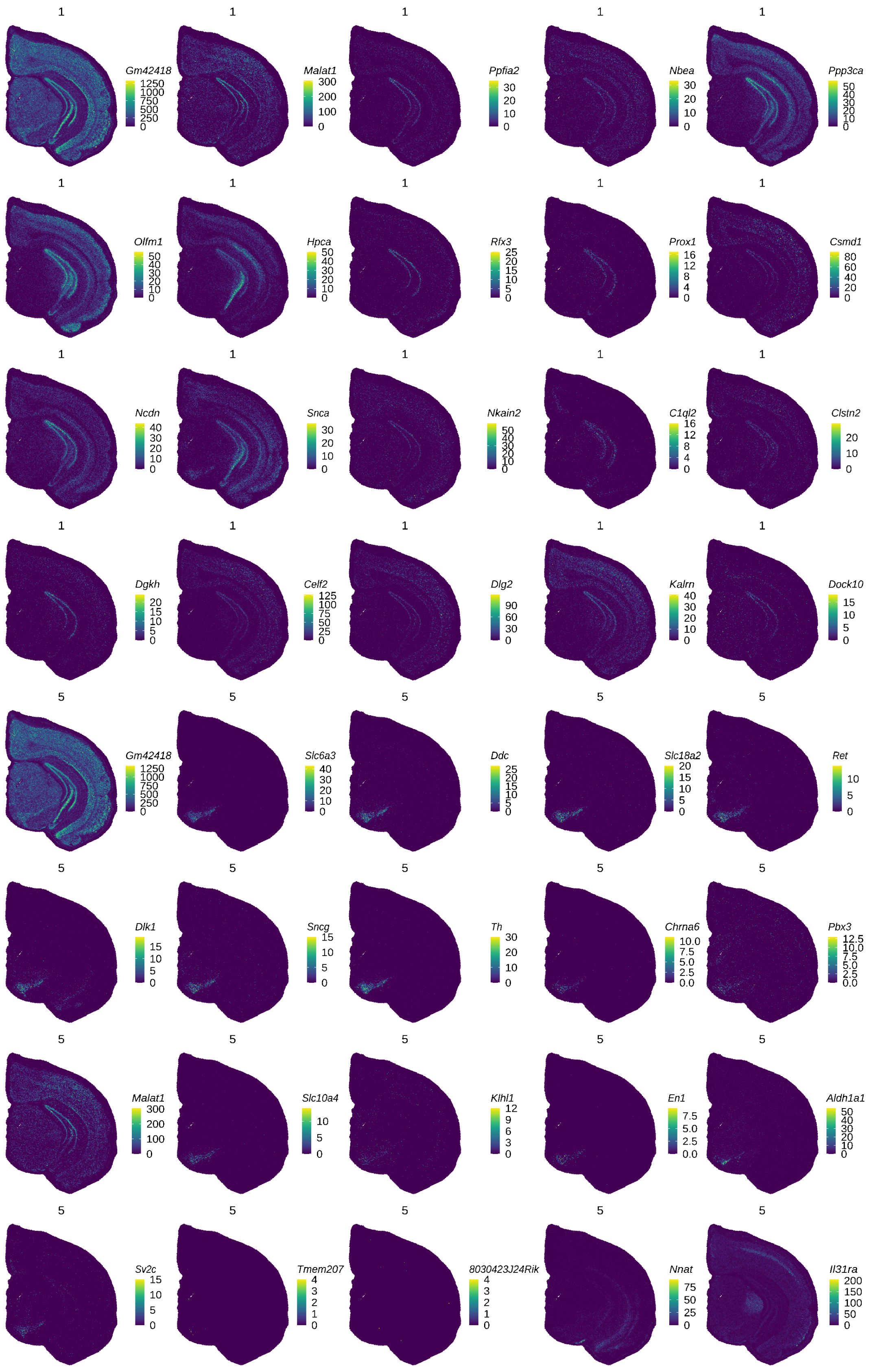

Fig. S5: Top 20 SVGs of cluster 1 and 5 for mouse brain dataset from Stereo-seq technology.

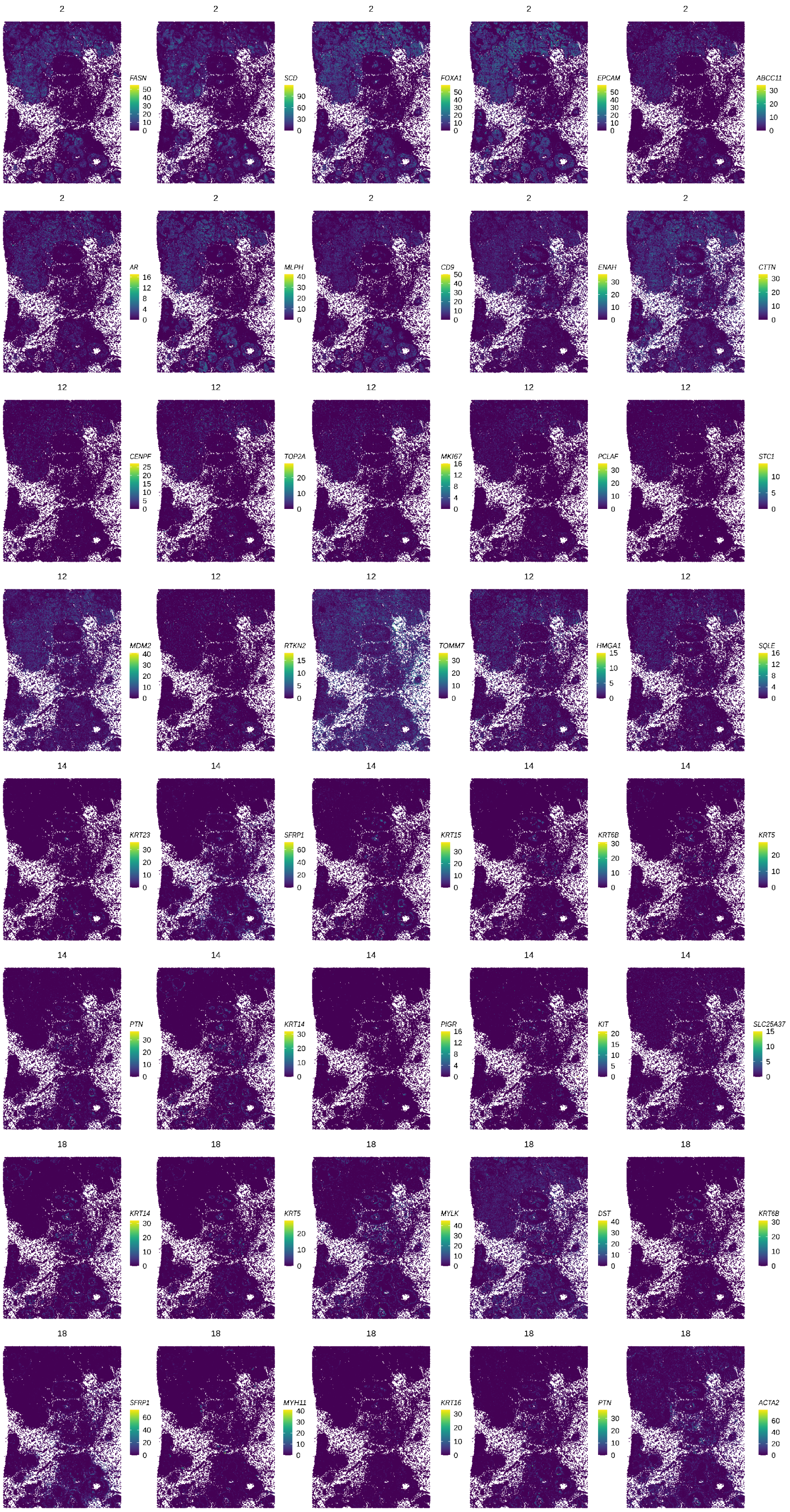

Fig. S6: Top 10 SVGs of cluster 2, 12, 14 and 18 for human breast cancer dataset from 10x Xenium technology.

Running time comparison on datasets sampled from the NSCLC dataset from CosMx technology

Running time (hour)

Unit number	stDyer-image	SpaGCN	stLearn	SiGra	DeepST	MUSE
100,000	0.80	0.98	7.30	1.17	-	3.63
200,000	1.85	4.38	23.36	-	-	6.70
500,000	5.25	-	-	-	-	15.63
1,000,000	17.06	-	-	-	-	-

Fig. S7: The running time of methods that can utilize images.

Filtering biologically meaningful clusters

Spatially variable genes are identified for each cluster and can aid in filtering out clusters that are not biologically meaningful. For example, the cluster 16 predicted by stDyer-image (Figure S8) in the mouse brain dataset from Stereo-seq did not have a biologically meaningful shape. Users can check the SVGs associated with the cluster 16 (Figure S9) to judge whether the cluster 16 is biologically meaningful or not. For the cluster 16, there was no SVG uniquely highly expressed in the cluster among the top 20 SVGs. Only *Calb2*, *Calca* and *Pth2* were highly expressed in a broader or smaller region compared to the cluster 16.

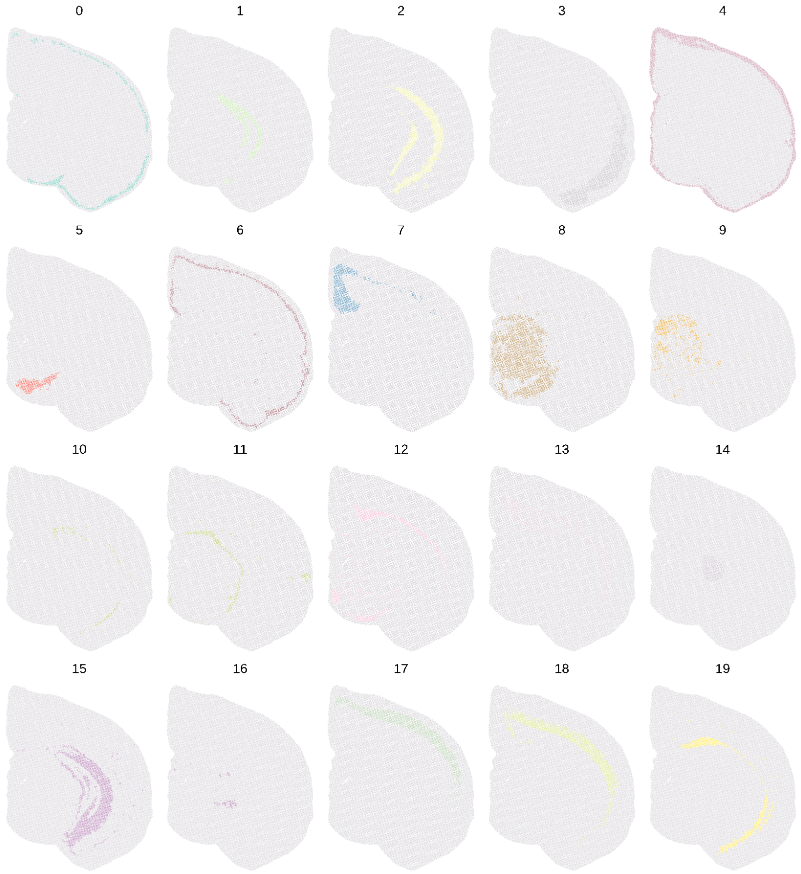

Fig. S8: The spatial domains of Mouse brain dataset from Stereo-seq predicted by stDyer-image.

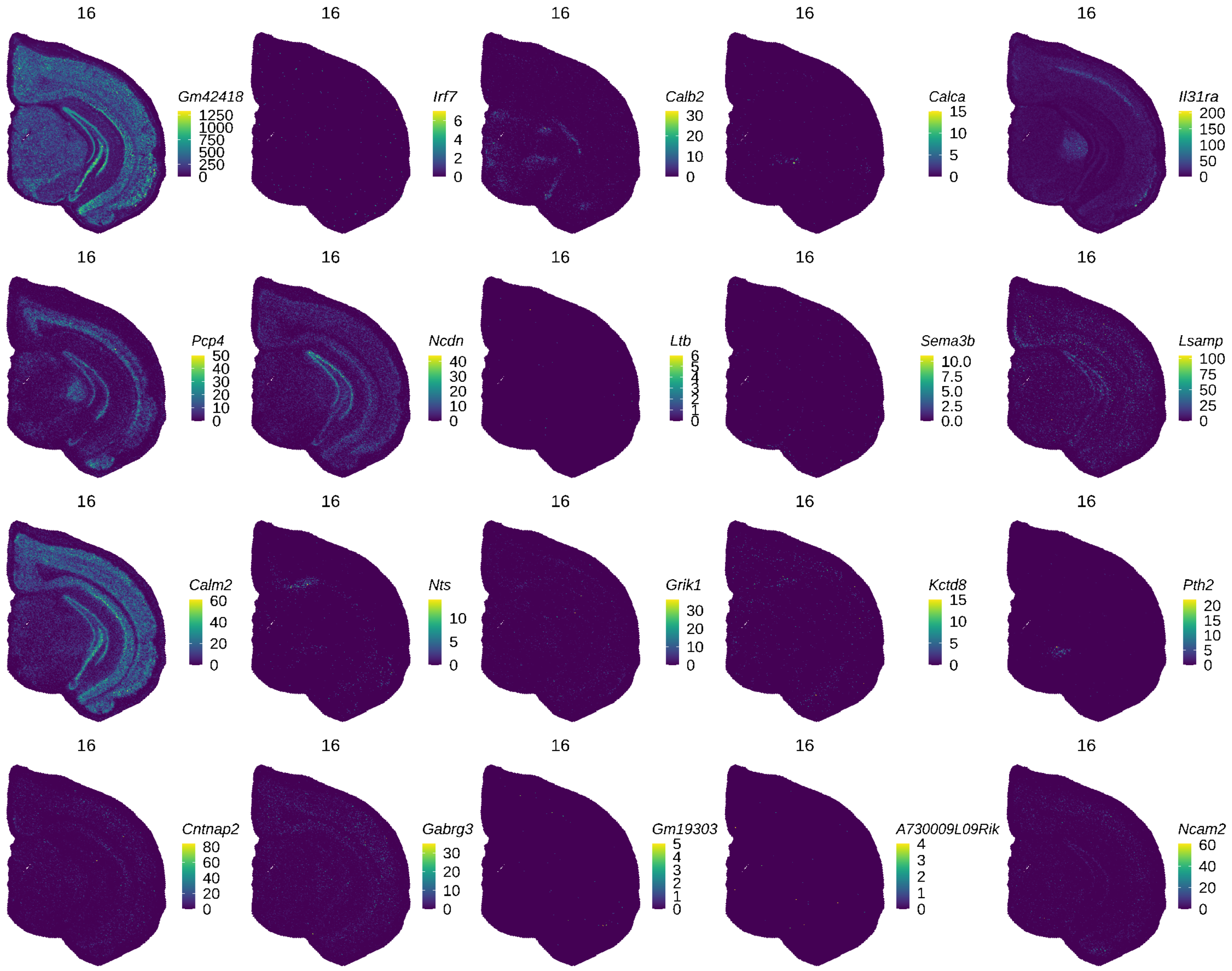

Fig. S9: The spatially variable genes associated with the predicted cluster 16 of stDyer-image on Mouse brain dataset from Stereo-seq.

Supplementary Methods

Multi-slice joint analysis

There are two scenarios for analyzing multiple slices jointly using stDyer-image. The first scenario involves a unified spatial coordinate system across all slices, while the second scenario involves independent spatial coordinate systems for each slice. Most datasets can be processed or directly fall into the first scenario. However, spatial coordinates of some public datasets may not be unified across slices due to a lack of information from dataset providers. In such cases, the spatial coordinates between slices may not reflect their true relative positions and can degrade the performance of stDyer-image for utilizing spatial coordinates to identify neighbors in spatial domain clustering. Under such circumstances, one way to mitigate the negative effects from unregistered spatial coordinates is to adjust the spatial coordinates of each slice to keep it far from the others. In this way, the neighbors of a unit obtained from spatial coordinates will always be within the same slice, avoiding being interfered with incorrect inter-slice spatial information. Meanwhile, the neighbors of a unit identified through similar image patches can be drawn from all slices and are not limited to the single slice where the unit locates.

Evaluation Metrics

The Silhouette score (Rousseeuw, 1987) is used to evaluate 1. the separateness of clustering results given the predicted cluster labels of each method and the gene expression; and 2. the separateness of embeddings given the learned embeddings of each method and the annotated labels. A higher SI score indicates better separateness, while a lower SI score indicates worse separateness. The SI score is computed as the average SI coefficients of all units. The SI coefficient is computed as follows:

$$s = \frac{b - a}{max(a, b)} \tag{S1}$$

where a is the average distance between a unit and all other units in the same cluster, and b is the average distance between a unit and all other units in the nearest cluster.

The Fowlkes-Mallows index (FMI) (Mallows and Fowlkes, 2012) measures whether pairs of units are consistently assigned to the same cluster in both predicted and true labels. It is defined as the geometric mean of precision and recall. A high FMI indicates a high precision and a high recall simultaneously. A high precision with a low recall or vice versa will lead to a low FMI.

$$FMI = \sqrt{Precision \cdot Recall} = \sqrt{\frac{TP}{TP + FP} \cdot \frac{TP}{TP + FN}} \tag{S2}$$

where TP, FP and FN denote true positive (the number of pairs of units that share the same cluster label in both predicted and true labels), false positive (the number of pairs of units that share the same cluster label in predicted labels but not in true labels) and false negative (the number of pairs of units that share the same cluster label in true labels but not in predicted labels), respectively.

Integrated gradient analysis

We incorporated the integrated gradient analysis (Sundararajan et al., 2017) in stDyer-image to identify SVGs. Briefly, it is a technique to estimate the importance of a gene for a model to predict the cluster label of a unit. We followed the same procedure as stDyer and the details of integrated gradient analysis can be referred to Xu et al. (2025).

Hyper-parameter settings

We set the learning rate to 0.001 empirically to balance the learning speed and stability. The batch sizes were set to 8,192, 4,096, 2,048 and 1,024 for the NSCLC dataset, human breast cancer dataset, human intestine dataset and mouse brain dataset, respectively. Large batch size is preferred when the GPU has enough memory and the dataset itself is large to enable faster training.

Comparison with other clustering methods

stDyer We set the weight of the image loss to zero to obtain the stDyer model. Other hyper-parameters were the same as stDyer-image.

SpaGCN We followed its tutorial to perform clustering analysis. Mclust was used to ensure the predicted cluster numbers were consistent with the annotated cluster numbers. The parameter "histology" is set to True and False to obtain the graphs that are used for clustering analysis with image information and refinement as recommended, respectively.

BayesSpace We followed its tutorial to perform clustering analysis. 2,000 highly variable features and 20 principal components were used as recommended by the tutorial if the feature number is more than 2,000. Otherwise, all features will be used followed by principal component analysis.

stLearn We followed its tutorial to perform clustering analysis. The parameter "n_comps" is set to 50 or the feature number if the feature number is less than 50 as recommended by the tutorial. "SME_normalize" is utilized to incorporate image information.

SiGra We followed its tutorial to perform clustering analysis.

MUSE We followed its tutorial to perform clustering analysis. The "latent_dim" is set to 100 or the feature number if the feature number is less than 100 as recommended by the tutorial.

DeepST We followed its tutorial to perform clustering analysis. Either 200 or the feature number the feature number is less than 200 of principal components were used to train DeepST for 1,000 epochs as recommended. The parameter "use_morphological" is set to True to utilize image information.

CellCharter We followed its tutorial to perform clustering analysis.

References

A. Janesick et al. High resolution mapping of the tumor microenvironment using integrated single-cell, spatial and in situ analysis. *Nature Communications*, 14(1):8353, 2023. ISSN 2041-1723. doi: 10.1038/s41467-023-43458-x.

C. L. Mallows et al. A method for comparing two hierarchical clusterings. *Taylor & Francis*, 2012.

S. L. O'Brien et al. Cenp-f expression is associated with poor prognosis and chromosomal instability in patients with primary breast cancer. *International Journal of Cancer*, 120(7):1434–1443, 2007. ISSN 1097-0215. doi: 10.1002/ijc.22413.

P. J. Rousseeuw. Silhouettes: A graphical aid to the interpretation and validation of cluster analysis. *Journal of Computational and Applied Mathematics*, 20:53–65, 1987. ISSN 0377-0427. doi: 10.1016/0377-0427(87)90125-7.

J. Sun et al. Overexpression of cenpf correlates with poor prognosis and tumor bone metastasis in breast cancer. *Cancer Cell International*, 19(1):264, 2019. ISSN 1475-2867. doi: 10.1186/s12935-019-0986-8.

M. Sundararajan et al. Axiomatic attribution for deep networks. In *Proceedings of the 34th International Conference on Machine Learning - Volume 70*, ICML'17, pages 3319–3328. JMLR.org, 2017. doi: 10.5555/3305890.3306024.

D. Vanauberg et al. Involvement of the pro-oncogenic enzyme fatty acid synthase in the hallmarks of cancer: a promising target in anti-cancer therapies. *Oncogenesis*, 12(1):16, 2023. ISSN 2157-9024. doi: 10.1038/s41389-023-00460-8.

K. Xu et al. stdyer enables spatial domain clustering with dynamic graph embedding. *Genome Biology*, 26(1):34, 2025. ISSN 1474-760X. doi: 10.1186/s13059-025-03503-y.

S. Xu et al. Fatty acid synthase promotes breast cancer metastasis by mediating changes in fatty acid metabolism. *Oncology Letters*, 21(1):27, 2021. ISSN 1792-1074. doi: 10.3892/ol.2020.12288.
